# Supplementary figures and images for: Virus-host co-evolution under a modified nuclear genetic code
Source: PeerJ. 2013 Mar 5;1:e50. doi: 10.7717/peerj.50 (PMC3628385; doi:10.7717/peerj.50)

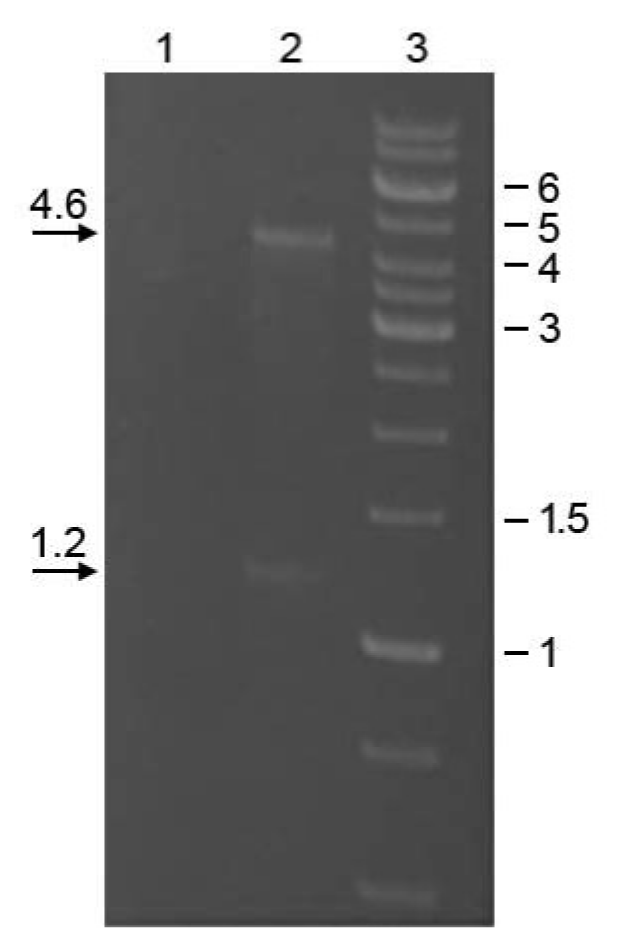

Supplement: Fig. S1 — Sample treated (lane 1) or untreated (lane 2) with an RNAse cocktail. Lane 3 is a DNA size marker labeled in kilobases. The gel was electrophoresed on a 1.4% agarose gel and stained with ethidium bromide. [file peerj-01-50-s001.png]

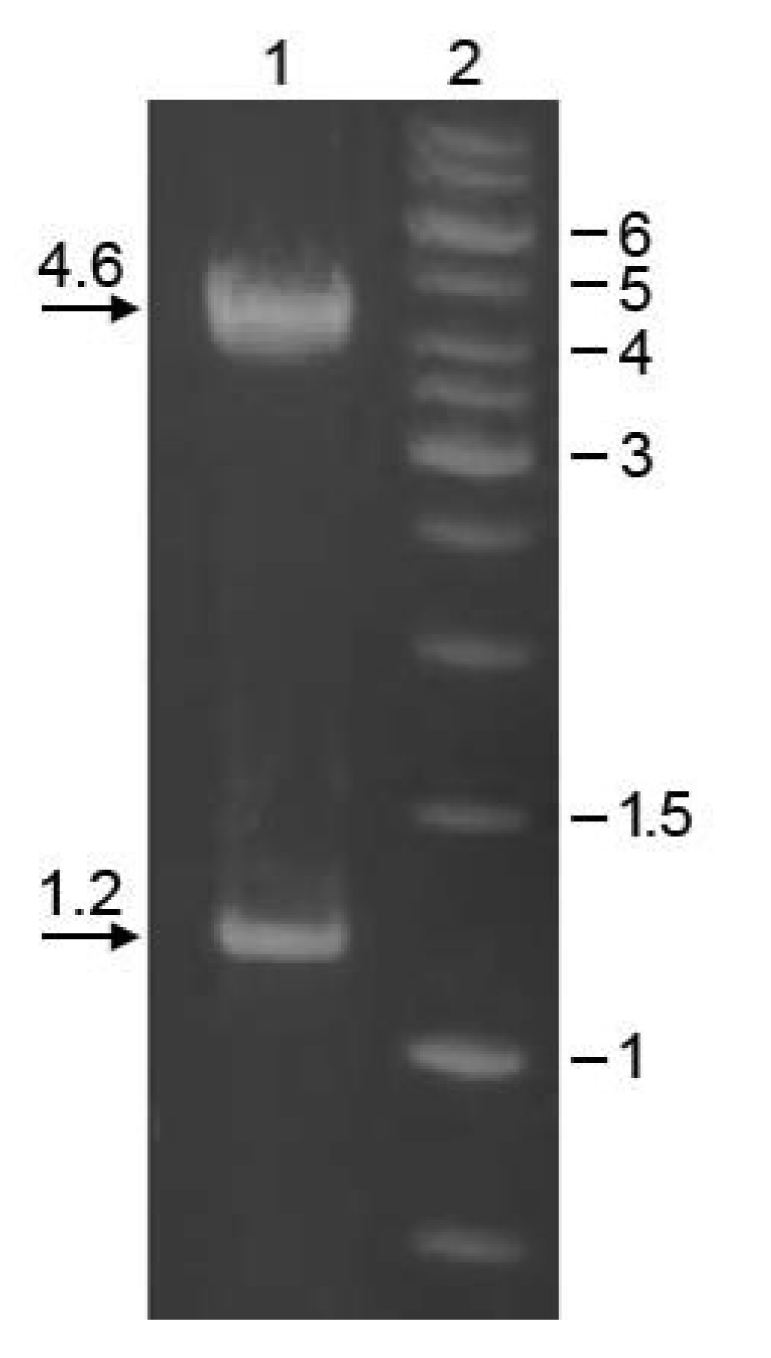

Supplement: Fig. S2 — The first lane shows the total nucleic acids extracted from CsCl purified viral particles from S. segobiensis of density 1.40 g/cc, comprised of the same nucleic acid band sizes (L and M) isolated by dsRNA CF11 chromatography. The DNA marker in lane 2 is labeled in kilobases. [file peerj-01-50-s002.png]

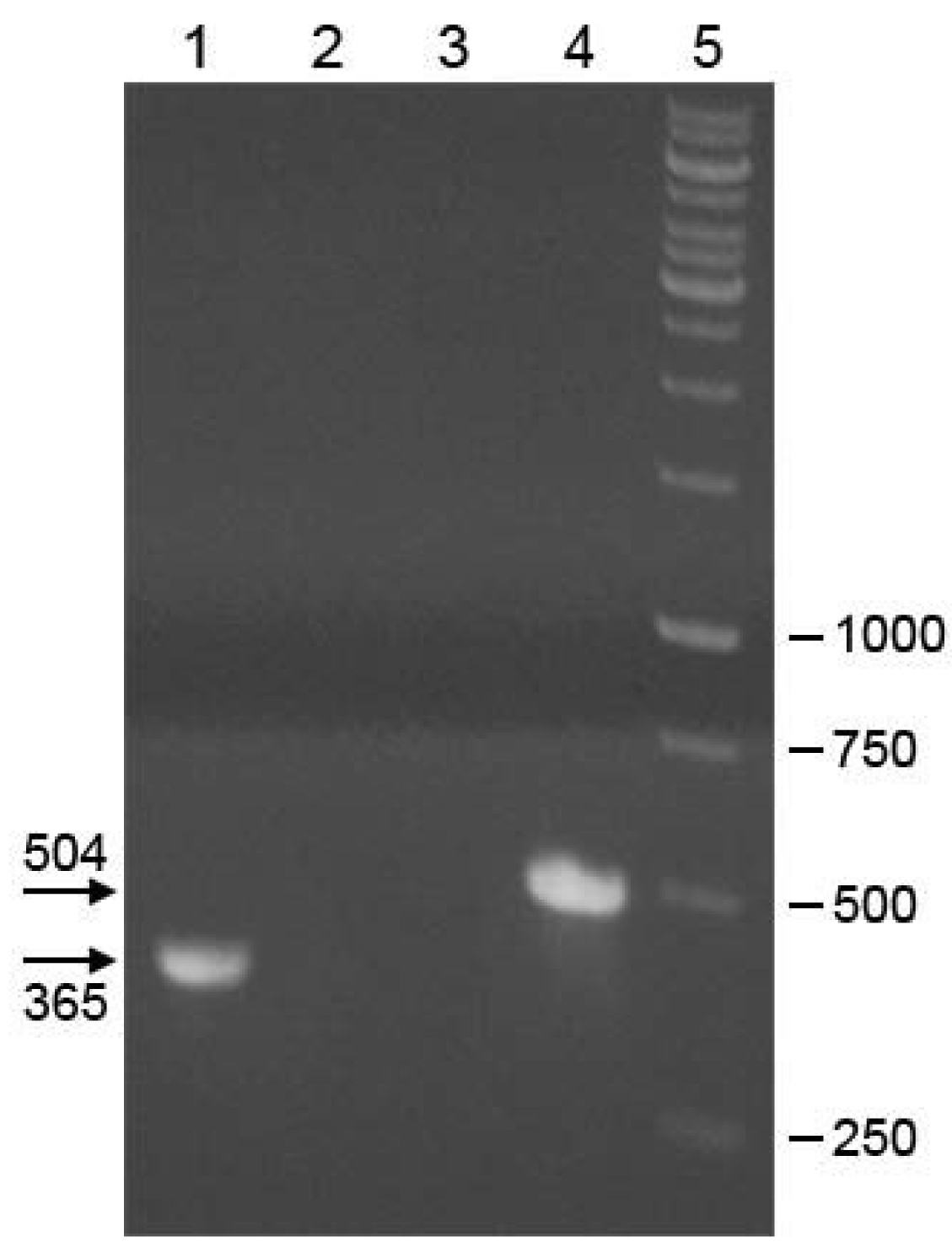

Supplement: Fig. S4 — Lane 1 contains M-MLV reverse transcriptase (RT), Taq polymerase, DNase-treated nucleic acids from S. segobiensis, and primers targeting a 365 bp region of the RdRp gene from the viral RNA. Lane 2 contains RT, Taq polymerase, no nucleic acid template, and the viral RdRp primers used in lane 1. Lane 3 contains Taq polymerase, total nucleic acid from S. segobiensis, and the same primers as lanes 1 and 2. This is a control against amplification of the RdRp target from the genomic DNA of S. segobiensis. Lane 4 contains Taq polymerase, whole nucleic acid from S. segobiensis, and primers targeting a 504 bp region of the host gene XYL1, xylose reductase. Lane 5 contains DNA markers. Band size labels are in base pairs. [file peerj-01-50-s004.png]
