# Supplementary material for: Virus-host co-evolution under a modified nuclear genetic code
Source: PeerJ. 2013 Mar 5;1:e50. doi: 10.7717/peerj.50 (PMC3628385; doi:10.7717/peerj.50)
Supplement: Table S1 — Fungal strains and NCBI accession numbers used for isolating protein sequences for the bioinformatics analyses of fungi. [file peerj-01-50-s007.docx]

| Species | MCM7 | ELP3 | KOG1 | GDH2 | ILV2 |
| --- | --- | --- | --- | --- | --- |
| *Ashbya gossypii* ATCC 10895 | [NP_984137](http://www.ncbi.nlm.nih.gov/protein/45187914?report=genbank&log$=protalign&blast_rank=4&RID=HU5U0YCY013) | [NP_986867](http://www.ncbi.nlm.nih.gov/protein/45201297?report=genbank&log$=protalign&blast_rank=7&RID=HU5U0YCY013) | [NP_984427](http://www.ncbi.nlm.nih.gov/protein/302307709?report=genbank&log$=protalign&blast_rank=1&RID=HU5U0YCY013) | [NP_986626](http://www.ncbi.nlm.nih.gov/protein/45201056?report=genbank&log$=protalign&blast_rank=2&RID=HU5U0YCY013) | [NP_984555](http://www.ncbi.nlm.nih.gov/protein/45190301?report=genbank&log$=protalign&blast_rank=5&RID=HU5U0YCY013) |
| *Candida albicans* SC5314 | [XP_712587](http://www.ncbi.nlm.nih.gov/protein/68487053?report=genbank&log$=protalign&blast_rank=6&RID=HU6CHASR01R) | [XP_716628](http://www.ncbi.nlm.nih.gov/protein/68478719?report=genbank&log$=protalign&blast_rank=10&RID=HU6CHASR01R) | [XP_715903](http://www.ncbi.nlm.nih.gov/protein/68480185?report=genbank&log$=protalign&blast_rank=1&RID=HU6CHASR01R) | [XP_715974](http://www.ncbi.nlm.nih.gov/protein/68480100?report=genbank&log$=protalign&blast_rank=3&RID=HU6CHASR01R) | [XP_721692](http://www.ncbi.nlm.nih.gov/protein/68468265?report=genbank&log$=protalign&blast_rank=8&RID=HU6CHASR01R) |
| *Candida dubliniensis* CD36 | [XP_002418794](http://www.ncbi.nlm.nih.gov/protein/241952144?report=genbank&log$=protalign&blast_rank=3&RID=HU6HUC8D013) | [XP_002419503](http://www.ncbi.nlm.nih.gov/protein/241953563?report=genbank&log$=protalign&blast_rank=5&RID=HU6HUC8D013) | [XP_002417184](http://www.ncbi.nlm.nih.gov/protein/241948923?report=genbank&log$=protalign&blast_rank=1&RID=HU6HUC8D013) | [XP_002418689](http://www.ncbi.nlm.nih.gov/protein/241951934?report=genbank&log$=protalign&blast_rank=2&RID=HU6HUC8D013) | [XP_002419168](http://www.ncbi.nlm.nih.gov/protein/241952893?report=genbank&log$=protalign&blast_rank=4&RID=HU6HUC8D013) |
| *Candida glabrata* CBS 138 | [XP_449582](http://www.ncbi.nlm.nih.gov/protein/50294341?report=genbank&log$=protalign&blast_rank=3&RID=HU6S4VWT01R) | [XP_449624](http://www.ncbi.nlm.nih.gov/protein/50294426?report=genbank&log$=protalign&blast_rank=5&RID=HU6S4VWT01R) | [XP_449926](http://www.ncbi.nlm.nih.gov/protein/50295030?report=genbank&log$=protalign&blast_rank=1&RID=HU6S4VWT01R) | [XP_446609](http://www.ncbi.nlm.nih.gov/protein/50288361?report=genbank&log$=protalign&blast_rank=2&RID=HU6S4VWT01R) | [XP_448375](http://www.ncbi.nlm.nih.gov/protein/50291885?report=genbank&log$=protalign&blast_rank=4&RID=HU6S4VWT01R) |
| *Candida orthopsilosis* Co 90-125 | [XP_003868687](http://www.ncbi.nlm.nih.gov/protein/448522428?report=genbank&log$=protalign&blast_rank=3&RID=HU6ZTD2M013) | [XP_003868521](http://www.ncbi.nlm.nih.gov/protein/448521569?report=genbank&log$=protalign&blast_rank=5&RID=HU6ZTD2M013) | [XP_003868026](http://www.ncbi.nlm.nih.gov/protein/448519014?report=genbank&log$=protalign&blast_rank=1&RID=HU6ZTD2M013) | [XP_003867686](http://www.ncbi.nlm.nih.gov/protein/448516991?report=genbank&log$=protalign&blast_rank=2&RID=HU6ZTD2M013) | [XP_003868842](http://www.ncbi.nlm.nih.gov/protein/448523041?report=genbank&log$=protalign&blast_rank=4&RID=HU6ZTD2M013) |
| *Candida parapsilosis* CDC317 | [CABE01000024](http://www.ncbi.nlm.nih.gov/nucleotide/218176148?report=genbank&log$=nuclalign&blast_rank=2&RID=HU7BWJ1S015) | [CABE01000008](http://www.ncbi.nlm.nih.gov/nucleotide/218176164?report=genbank&log$=nuclalign&blast_rank=3&RID=HU7BWJ1S015) | [CABE01000015](http://www.ncbi.nlm.nih.gov/nucleotide/218176157?report=genbank&log$=nuclalign&blast_rank=1&RID=HU7BWJ1S015) | [CABE01000015](http://www.ncbi.nlm.nih.gov/nucleotide/218176157?report=genbank&log$=nuclalign&blast_rank=1&RID=HU7BWJ1S015) | [CABE01000024](http://www.ncbi.nlm.nih.gov/nucleotide/218176148?report=genbank&log$=nuclalign&blast_rank=2&RID=HU7BWJ1S015) |
| *Candida tenuis* ATCC | [EGV59938](http://www.ncbi.nlm.nih.gov/protein/344228052?report=genbank&log$=protalign&blast_rank=3&RID=HU7CPKVG013) | [EGV65912](http://www.ncbi.nlm.nih.gov/protein/344234042?report=genbank&log$=protalign&blast_rank=7&RID=HU7CPKVG013) | [EGV66317](http://www.ncbi.nlm.nih.gov/protein/344234447?report=genbank&log$=protalign&blast_rank=1&RID=HU7CPKVG013) | [EGV61770](http://www.ncbi.nlm.nih.gov/protein/344229885?report=genbank&log$=protalign&blast_rank=2&RID=HU7CPKVG013) | [EGV61074](http://www.ncbi.nlm.nih.gov/protein/344229188?report=genbank&log$=protalign&blast_rank=5&RID=HU7CPKVG013) |
| *Candida tropicalis* MYA-3404 | [XP_002547663](http://www.ncbi.nlm.nih.gov/protein/255725468?report=genbank&log$=protalign&blast_rank=3&RID=HU7KT6M901R) | [XP_002547960](http://www.ncbi.nlm.nih.gov/protein/255726068?report=genbank&log$=protalign&blast_rank=5&RID=HU7KT6M901R) | [XP_002550249](http://www.ncbi.nlm.nih.gov/protein/255730649?report=genbank&log$=protalign&blast_rank=1&RID=HU7KT6M901R) | [XP_002547366](http://www.ncbi.nlm.nih.gov/protein/255724874?report=genbank&log$=protalign&blast_rank=2&RID=HU7KT6M901R) | [XP_002548114](http://www.ncbi.nlm.nih.gov/protein/255726376?report=genbank&log$=protalign&blast_rank=4&RID=HU7KT6M901R) |
| *Debaryomyces hansenii* CBS 767 | [XP_457383](http://www.ncbi.nlm.nih.gov/protein/50414232?report=genbank&log$=protalign&blast_rank=3&RID=HU7TE79G01R) | [XP_459174](http://www.ncbi.nlm.nih.gov/protein/50421257?report=genbank&log$=protalign&blast_rank=5&RID=HU7TE79G01R) | [XP_459649.2](http://www.ncbi.nlm.nih.gov/protein/294657333?report=genbank&log$=protalign&blast_rank=1&RID=HU7TE79G01R) | [XP_458121.2](http://www.ncbi.nlm.nih.gov/protein/294655902?report=genbank&log$=protalign&blast_rank=2&RID=HU7TE79G01R) | [XP_461633.2](http://www.ncbi.nlm.nih.gov/protein/294659277?report=genbank&log$=protalign&blast_rank=4&RID=HU7TE79G01R) |
| *Kluyveromyces lactis* NRRL Y-1140 | [XP_454998](http://www.ncbi.nlm.nih.gov/protein/50309969?report=genbank&log$=protalign&blast_rank=3&RID=HU8084NG015) | [XP_455030](http://www.ncbi.nlm.nih.gov/protein/50310033?report=genbank&log$=protalign&blast_rank=5&RID=HU8084NG015) | [XP_451414](http://www.ncbi.nlm.nih.gov/protein/50302953?report=genbank&log$=protalign&blast_rank=1&RID=HU8084NG015) | [XP_453440](http://www.ncbi.nlm.nih.gov/protein/50306929?report=genbank&log$=protalign&blast_rank=2&RID=HU8084NG015) | [XP_452091](http://www.ncbi.nlm.nih.gov/protein/50304283?report=genbank&log$=protalign&blast_rank=4&RID=HU8084NG015) |
| *Komagataella pastoris* GS115 | XP_002494340 | XP_002493503 | XP_002491366 | XP_002491206 | XP_002493637 |
| *Lodderomyces elongisporus* NRRL YB-4239 | [XP_001526012](http://www.ncbi.nlm.nih.gov/protein/149240273?report=genbank&log$=protalign&blast_rank=5&RID=HU84AK8E013) | [XP_001526261](http://www.ncbi.nlm.nih.gov/protein/149241028?report=genbank&log$=protalign&blast_rank=4&RID=HU84AK8E013) | [XP_001526910](http://www.ncbi.nlm.nih.gov/protein/149244734?report=genbank&log$=protalign&blast_rank=1&RID=HU84AK8E013) | [XP_001527104](http://www.ncbi.nlm.nih.gov/protein/149245140?report=genbank&log$=protalign&blast_rank=2&RID=HU84AK8E013) | [XP_001525930](http://www.ncbi.nlm.nih.gov/protein/149240109?report=genbank&log$=protalign&blast_rank=3&RID=HU84AK8E013) |
| *Millerozyma farinosa* CBS 7064 | XP_004200401 | XP_004199480 | XP_004197044 | XP_004195806 | XP_004204601 |
| *Naumovozyma castellii* CBS 4309 | XP_003674682 | XP_003674663 | XP_003677044 | XP_003675984 | XP_003676989 |
| *Pichia angusta* | [AEOI01000007](http://www.ncbi.nlm.nih.gov/nucleotide/320582261?report=genbank&log$=nuclalign&blast_rank=3&RID=HU8D2DRE01R) | [AEOI01000003](http://www.ncbi.nlm.nih.gov/nucleotide/320583369?report=genbank&log$=nuclalign&blast_rank=1&RID=HU8D2DRE01R) | [AEOI01000003](http://www.ncbi.nlm.nih.gov/nucleotide/320583369?report=genbank&log$=nuclalign&blast_rank=1&RID=HU8D2DRE01R) | [AEOI01000008](http://www.ncbi.nlm.nih.gov/nucleotide/320582046?report=genbank&log$=nuclalign&blast_rank=2&RID=HU8D2DRE01R) | [AEOI01000012](http://www.ncbi.nlm.nih.gov/nucleotide/320580434?report=genbank&log$=nuclalign&blast_rank=4&RID=HU8D2DRE01R) |
| *Saccharomyces bayanus* 623-6C YM4911 | AACG02000001 | AACG02000030 | AACG02000047 | AACG02000151 | AACG02000014 |
| *Saccharomyces cerevisiae* S288c | NP_009761.4 | NP_015239 | NP_012056 | NP_010066 | NP_013826 |
| *Saccharomyces mikatae* IFO 1815 | AABZ01000121 | AACH01000557 | AABZ01000023 | AABZ01000088 | AACH01000297 |
| *Saccharomyces paradoxus* NRRL Y-17217 | AABY01000082 | AABY01000069 | AABY01000061 | AABY01000002 | AABY01000053 |
| *Scheffersomyces coipomoensis* NRRL Y-17651(this study) | KC616425 | KC616424 | KC616426 | KC616427 | KC616428 |
| *Scheffersomyces segobiensis* NRL Y-11571(this study) | KC616420 | KC616421 | KC616422 | KC616423 | KC616419 |
| *Scheffersomyces stipitis* CBS 6054 | XP_001384179 | XP_001382617.2 | XP_001387441.2 | XP_001384885 | XP_001384614.2\| |
| *Spathaspora passalidarum* NRRL Y-27907 | EGW32845 | EGW31709 | EGW35348 | EGW33006 | EGW31477 |
