# Supplementary material for: Virus-host co-evolution under a modified nuclear genetic code
Source: PeerJ. 2013 Mar 5;1:e50. doi: 10.7717/peerj.50 (PMC3628385; doi:10.7717/peerj.50)
Supplement: Table S2 [file peerj-01-50-s008.docx]

| **Organism** | **CTG RSCU** | **CTC RSCU** | **CTA RSCU** | **TTG RSCU** | **TTA RSCU** | **%GC** | **%G3C3** | **%C** | **%C3** | **%G** | **%G3** | **%A3** | **Code** |
| --- | --- | --- | --- | --- | --- | --- | --- | --- | --- | --- | --- | --- | --- |
| Amasya cherry disease associated mycovirus | 1.795 | 0.359 | 1.026 | 1.846 | 0.462 | 49.47 | 51.78 | 20.8 | 21.08 | 28.67 | 33.98 | 26.64 | VIR |
| Aspergillus foetidus slow virus | 1.367 | 1.633 | 0.722 | 0.759 | 0.38 | 57.87 | 61.36 | 30.13 | 37.56 | 27.74 | 24.94 | 12.85 | VIR |
| Aspergillus mycovirus | 1.807 | 1.012 | 0.578 | 1.012 | 0.723 | 53.98 | 57.02 | 26.7 | 31.24 | 27.28 | 27.5 | 16.15 | VIR |
| Beauveria bassiana virus 1 | 1.078 | 1.617 | 0.719 | 1.186 | 0.611 | 55.65 | 56.35 | 28.57 | 33.48 | 27.09 | 23.92 | 15.68 | VIR |
| Black raspberry virus F | 1.615 | 0.323 | 1.154 | 1.246 | 1.108 | 45.81 | 43.71 | 19.06 | 18.09 | 26.75 | 28.06 | 28 | VIR |
| Botryotinia fuckeliana totivirus 1(133919431) | 1.012 | 1.554 | 0.759 | 0.94 | 0.506 | 54.83 | 58.02 | 28.48 | 34.39 | 26.36 | 24.79 | 18.97 | VIR |
| Candida albicans | 0.16 | 0.07 | 0.02 | 2.27 | 2.36 | 36.18 | 24.48 | 16.4 | 13.52 | 19.79 | 12.87 | 31.6 | CTG |
| Candida dubliniensis | 0.16 | 0.06 | 0.09 | 2.22 | 2.35 | 35.94 | 23.23 | 16.22 | 12.57 | 19.72 | 12.61 | 33.13 | CTG |
| Candida glabrata | 0.98 | 0.06 | 1 | 1.98 | 1.31 | 42.52 | 41.3 | 20.18 | 22 | 22.34 | 20.95 | 25.63 | STD |
| Candida orthopsilosis | 0.25 | 0.13 | 0.18 | 2.88 | 1.35 | 37.25 | 26.25 | 16.54 | 13.19 | 20.71 | 15.02 | 30.85 | CTG |
| Candida parapsilosis | 0.25 | 0.16 | 0.13 | 3.22 | 1.06 | 38.22 | 29.41 | 16.83 | 14.07 | 21.39 | 17.24 | 29.82 | CTG |
| Candida tenuis | 0.15 | 0.27 | 0.1 | 2.84 | 1.35 | 43.25 | 45.64 | 20.5 | 25.25 | 22.75 | 21.72 | 23.16 | CTG |
| Candida tropicalis | 0.1 | 0.04 | 0.04 | 2.38 | 2.37 | 34.96 | 20.34 | 15.97 | 12.95 | 18.99 | 9.4 | 31.89 | CTG |
| Cherry chlorotic rusty spot associated | 2.222 | 0.667 | 0.889 | 0.889 | 1.111 | 51.67 | 52.23 | 22.01 | 22.33 | 29.67 | 32.36 | 28.8 | VIR |
| Coniothyrium minitans mycovirus | 1.348 | 2.087 | 0.391 | 1 | 0.391 | 59.35 | 63.74 | 30.74 | 38.6 | 28.61 | 26.41 | 15.04 | VIR |
| Debaryomyces hansenii | 0.16 | 0.02 | 0.11 | 1.97 | 2.53 | 38.07 | 31.35 | 17.51 | 17.36 | 20.56 | 15.87 | 30.09 | CTG |
| Epichloe festucae virus 1 | 1.091 | 2.377 | 0.623 | 0.584 | 0.468 | 60.24 | 63.02 | 32.47 | 38.67 | 27.77 | 25.48 | 15.04 | VIR |
| Eremothecium gossypii | 1.47 | 0.88 | 0.78 | 1.89 | 0.46 | 52.97 | 65.72 | 24.44 | 30.12 | 28.53 | 36.77 | 13.81 | STD |
| Fusarium virguliforme mycovirus 2 | 0.9 | 0.923 | 1.085 | 1.038 | 0.9 | 50.98 | 47.97 | 26.64 | 26.72 | 24.34 | 22.98 | 21.77 | VIR |
| Gremmeniella abietina RNA virus L1 | 1.348 | 1.87 | 0.652 | 0.913 | 0.652 | 57.08 | 55.37 | 30.26 | 34.81 | 26.82 | 22.02 | 16.97 | VIR |
| Gremmeniella abietina RNA virus L2 | 1.034 | 1.655 | 0.621 | 0.966 | 0.828 | 54.24 | 54.22 | 28.13 | 33.29 | 26.11 | 22.64 | 19.37 | VIR |
| Helicobasidium mompa No 17 dsRNA virus | 1.066 | 1.5 | 1.066 | 0.987 | 0.355 | 55.64 | 57.24 | 26.63 | 31.87 | 29.01 | 27.03 | 17.92 | VIR |
| Helminthosporium victoriae 145S virus | 1.826 | 0.457 | 0.652 | 1.957 | 0.522 | 45.23 | 46.99 | 15.64 | 16.01 | 29.59 | 34.59 | 23.55 | VIR |
| Helminthosporium victoriae virus 190S | 0.8 | 1.8 | 0.6 | 1.28 | 0.52 | 58.32 | 61.51 | 30.52 | 37.54 | 27.8 | 25.05 | 13.92 | VIR |
| Kluyveromyces lactis | 0.24 | 0.28 | 0.92 | 2.73 | 1.38 | 40.77 | 37.2 | 19.49 | 20.48 | 21.29 | 18.39 | 26.53 | STD |
| Komagataella pastoris | 1.09 | 0.49 | 0.74 | 1.7 | 0.83 | 42.95 | 39.96 | 20.77 | 21.6 | 22.19 | 19.99 | 24.93 | STD |
| Lodderomyces elongisporus | 0.21 | 0.51 | 0.12 | 3.11 | 0.77 | 41.34 | 37.85 | 19.49 | 20.86 | 21.85 | 18.68 | 29.23 | CTG |
| Magnaporthe oryzae virus 1 | 1.222 | 2.148 | 0.63 | 0.852 | 0.185 | 58.48 | 59.75 | 32.64 | 36.9 | 25.84 | 23.92 | 11.14 | VIR |
| Magnaporthe oryzae virus 2 | 1.708 | 2 | 0.583 | 0.833 | 0.375 | 62.24 | 69.38 | 32.92 | 40.86 | 29.32 | 29.38 | 13.83 | VIR |
| Millerozyma farinosa | 0.24 | 0.37 | 0.15 | 2.45 | 1.38 | 41.9 | 40.31 | 19.2 | 21.09 | 22.71 | 20.93 | 25.74 | CTG |
| Naumovozyma castellii | 0.16 | 0.04 | 0.53 | 1.76 | 2.96 | 35.91 | 24.33 | 16.37 | 12.73 | 19.54 | 13.84 | 32.15 | STD |
| Ogataea angusta | 2 | 0.91 | 0.36 | 1.41 | 0.14 | 50.88 | 60.9 | 24.84 | 32.56 | 26.04 | 29.43 | 15.36 | STD |
| Phlebiopsis gigantea mycovirus dsRNA 2 | 0.635 | 1.314 | 0.964 | 0.635 | 1.314 | 50.68 | 44.21 | 29.12 | 29.6 | 21.56 | 16.68 | 21.08 | VIR |
| Phytophthora infestans RNA virus 3 | 1.282 | 0.627 | 0.655 | 1.991 | 0.545 | 55.2 | 56.32 | 24.72 | 25.59 | 30.48 | 32.59 | 16.15 | VIR |
| Rosellinia necatrix quadrivirus 1 | 2.293 | 0.537 | 1.61 | 0.927 | 0.488 | 52.02 | 55.39 | 23.19 | 26.24 | 28.83 | 31.43 | 25.32 | VIR |
| Saccharomyces bayanus | 0.86 | 0.25 | 0.96 | 2.11 | 1.41 | 41.37 | 38.75 | 20.2 | 22.23 | 21.17 | 18.3 | 26.47 | STD |
| Saccharomyces cerevisae virus L-A | 1.096 | 0.626 | 1.2 | 1.252 | 1.2 | 45.81 | 41.03 | 21.37 | 21.19 | 24.44 | 22.67 | 25.55 | VIR |
| Saccharomyces cerevisae virus L-BC(La) | 0.857 | 0.454 | 0.958 | 1.664 | 1.412 | 42.52 | 38.41 | 18.64 | 18.13 | 23.88 | 23.14 | 26.89 | VIR |
| Saccharomyces cerevisiae | 0.64 | 0.19 | 0.99 | 1.87 | 1.79 | 39.59 | 33.75 | 18.65 | 17.89 | 20.94 | 17.76 | 28.26 | STD |
| Saccharomyces mikatae | 0.69 | 0.22 | 0.88 | 1.9 | 1.8 | 39.41 | 33.71 | 18.97 | 18.93 | 20.44 | 16.63 | 28.87 | STD |
| Saccharomyces paradoxus | 0.55 | 0.16 | 1.12 | 1.75 | 1.92 | 39.52 | 33.62 | 18.88 | 18.67 | 20.63 | 16.85 | 28.54 | STD |
| Scheffersomyces coipomoensis | 0.06 | 0.04 | 0.02 | 0.53 | 3.77 | 32.55 | 14.16 | 15 | 9.27 | 17.55 | 6.96 | 34.44 | CTG |
| Scheffersomyces segobiensis | 0.1 | 0.49 | 0.13 | 3.35 | 0.57 | 45.5 | 50.72 | 21.8 | 29.11 | 23.7 | 22.97 | 20.72 | CTG |
| Scheffersomyces segobiensis virus | 0.06 | 0.123 | 0.205 | 2.959 | 1.932 | 37.8 | 27.69 | 13.62 | 6.4 | 24.18 | 23.8 | 25.87 | VIR |
| Scheffersomyces stipitis | 0.07 | 0.51 | 0.05 | 3.21 | 0.68 | 45.67 | 50.99 | 22.4 | 30.25 | 23.27 | 22.06 | 20.98 | CTG |
| Spathaspora passalidarum | 0.2 | 0.08 | 0.02 | 2.57 | 1.61 | 40.12 | 34.87 | 19.07 | 20.89 | 21.04 | 15.82 | 24.51 | CTG |
| Thielaviopsis basicola dsRNA virus 1 | 1.439 | 0.912 | 0.596 | 1.614 | 0.702 | 52.78 | 49.4 | 24.9 | 26.27 | 27.89 | 24.82 | 14.1 | VIR |
| Thielaviopsis basicola dsRNA virus 2 | 0.607 | 1.483 | 0.607 | 1.079 | 0.876 | 48.64 | 41.98 | 25.02 | 23.61 | 23.61 | 20.05 | 15.96 | VIR |
| Tolypocladium cylindrosporum virus 1 | 0.879 | 2.522 | 0.65 | 0.65 | 0.382 | 61.1 | 65.25 | 34.73 | 44.81 | 26.38 | 21.31 | 10.19 | VIR |
| Tolypocladium cylindrosporum virus 2 | 0.98 | 0.673 | 0.98 | 1.286 | 1.224 | 50.84 | 51.34 | 20.95 | 22.77 | 29.89 | 30.8 | 22.42 | VIR |
| Tuber aestivum virus 1 | 0.405 | 0.284 | 0.811 | 1.662 | 2.595 | 42.55 | 38.1 | 18.46 | 17.57 | 24.09 | 23.74 | 29.44 | VIR |
| Ustilago maydis virus UmVH1 | 1.31 | 1.207 | 0.931 | 1.069 | 0.828 | 51.76 | 52.79 | 25.36 | 27.56 | 26.4 | 27.45 | 24.8 | VIR |
| Xanthophyllomyces dendrorhous virus L1A | 1.5 | 0.214 | 1.393 | 1.179 | 1.179 | 44.09 | 46.13 | 19.03 | 22.18 | 25.07 | 26.51 | 32.94 | VIR |
| Xanthophyllomyces dendrorhous virus L1b | 1.475 | 0.508 | 0.814 | 1.475 | 1.22 | 45.16 | 46.06 | 19.86 | 23.12 | 25.3 | 25.69 | 29.19 | VIR |
| Xanthophyllomyces dendrorhous virus L2 | 1.348 | 0.472 | 1.079 | 1.146 | 1.079 | 45.22 | 45.55 | 20.39 | 22.51 | 24.83 | 25.69 | 30.79 | VIR |
